# Pseudotime dynamics of T cells in pancreatic ductal adenocarcinoma inform distinct functional states within the regulatory and cytotoxic T cells

**DOI:** 10.1016/j.isci.2023.106324

**Published:** 2023-03-07

**Authors:** Ashwin Jainarayanan, Nithishwer Mouroug-Anand, Edward H. Arbe-Barnes, Adam J. Bush, Rachael Bashford-Rogers, Adam Frampton, Lara Heij, Mark Middleton, Michael L. Dustin, Enas Abu-Shah, Shivan Sivakumar

**Affiliations:** 1 Kennedy Institute of Rheumatology, Nuffield Department of Orthopaedics, Rheumatology and Musculoskeletal Sciences, University of Oxford, OX3 7FY Oxford, UK; 2 Interdisciplinary Bioscience Doctoral Training Centre, University of Oxford, OX1 3NP Oxford, UK; 3 Department of Biochemistry, University of Oxford, OX1 3QU Oxford, UK; 4 University of Oxford Medical School, OX3 9DU Oxford, UK; 5 Wellcome Trust Centre for Human Genetics, University of Oxford, OX3 7BN Oxford, UK; 6 Department of Surgery, University of Surrey, Surrey, GU2 7XH Guildford, UK; 7 Department of Pathology, University of Aachen, 52062 Aachen, Germany; 8 Department of Oncology, University of Oxford, OX3 7DQ Oxford, UK; 9 Sir William Dunn School of Pathology, University of Oxford, OX1 3RE Oxford, UK

**Keywords:** Immune response, Bioinformatics, Cancer systems biology

## Abstract

Pancreatic ductal adenocarcinoma (PDAC) is among the deadliest types of cancer and has a 5-year survival of less than 8% owing to its complex biology. As PDAC is refractory to immunotherapy, we need to understand the functional dynamics of T cells in the PDAC microenvironment to develop alternative therapeutic strategies. In this study, we performed RNA velocity-based pseudotime analysis on a scRNA-seq dataset from surgically resected human PDAC specimens to gain insight into temporal gene expression patterns that best characterize the cell fates. The tumor microenvironment was seen to encompass a range of terminal states for the T cell trajectories with suppressive and non-tumor-responsive T cells dominating them. However, the results also reveal the existence of a functional branch of the T cell population that was not transitioning to exhausted and senescent states. These findings reveal various microenvironmental signals driving T cell patterns which can be useful in identifying new therapeutic avenues.

## Highlights

• PDAC is amongst the deadliest of cancers
• Understanding the cancer microenvironment is crucial to developing PDAC immunotherapy
• We utilized RNA velocity-based pseudotime analysis to characterize the cell fates
• Velocity trajectories reveal immune profiles with dysfunctional and functional states

## Graphical abstract

**Figure:**
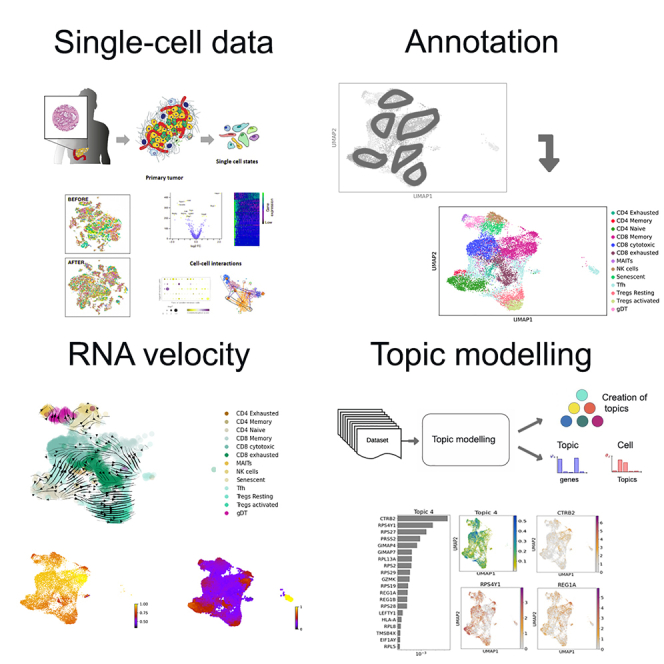

## Teaser

Immune respons; Bioinformatics; Cancer systems biology

## Introduction

Pancreatic ductal adenocarcinoma (PDAC) is one of the most aggressive forms of cancer, and the seventh leading cause of cancer-related death globally.^1^ Immune checkpoint inhibitors to PD1/PDL1, CTLA4, and CD40 have not shown clinical benefit in PDAC.^2^^,^^3^ Nonetheless, higher CD8^+^ T cell infiltration is associated with greater survival in PDAC.^4^^,^^5^^,^^6^ Single-cell technologies have further described T cell diversity in PDAC, including the presence of dysfunctional T cells and highly activated regulatory T cells (Tregs).^7^^,^^8^^,^^9^^,^^10^^,^^11^^,^^12^^,^^13^^,^^14^^,^^15^^,^^16^ Studying the pseudotemporal characterization of T cells subsets and functional states within the microenvironment might yield new insight and identify targets that can be leveraged in PDAC.

Temporal-omics in computational biology encompasses a variety of single-cell techniques used to observe the temporal genomic dynamics of an RNA-sequenced population. These techniques allow for the prediction of the future states of cells.^17^^,^^18^^,^^19^^,^^20^ One of the most prominent computational tools within temporal-omics is RNA velocity analysis. This technique was developed by La Manno et al. in 2018 to describe the rate of change of genetic expression within an RNA-sequenced cell population.^18^ Coupled first-order differential equations describe the relative abundances of spliced and unspliced mRNA. The future state of an RNA-sequenced cell can then be inferred by the ratio between the spliced and unspliced transcript readouts.

Two predominant variants of RNA velocity analyses exist. The original “velocyto” method requires partial estimation of a steady state in expression kinetics to estimate RNA velocity, whereas the later likelihood-based “scVelo” dynamical approach can estimate velocities within transient systems.^18^^,^^21^ While a recent technological development, RNA velocity analysis has seen a range of research applications. Notably, the ability of the tool to elucidate developmental lineages and cellular dynamics has been applied to infection monitoring, embryonic development, and the progression of chronic diseases, such as cancer.^22^

As an example, Krieger et al. recently employed RNA velocity analysis to investigate genomic heterogeneity in PDAC and observed distinct pathways for cellular differentiation in organoids and their associated metastases.^23^ Recent temporal-omics work in immuno-oncology includes the inference of the migratory direction of Tregs in lymphoid and barrier tissues, as a component of a novel pan-cancer T cell atlas and predicting the trajectories of developmental lineages for CD8^+^ T cells in B16 melanoma and MC38 colorectal adenocarcinoma murine models.^24^^,^^25^^,^^26^ Van Braeckel-Budimir et al. also observed the variation in T cell phenotype following combinatorial immune checkpoint blockade (ICB) immunotherapy consisting of anti-PD-L1 or anti-PD1 with costimulatory anti-OX-40 and anti-4-1BB agents. Using RNA velocity analysis, upregulation of CD8+ activation-associated transcription factors and recruitment of “stem-like”-activated CD8^+^ cells from secondary lymphoid tissues were reported in both partially ICB-responsive MC38 and the ICB-resistant B16 melanoma murine models.^26^

Topic models are statistical methods for discovering the abstract themes that pervade a large and seemingly unstructured dataset. These models are highly efficient in organizing the dataset with the topics learned and are extensively used for natural language processing datasets. Here, we use Latent Dirichlet Allocation (LDA), a topic model to analyze the single-cell RNA-sequencing (scRNA-seq) dataset and infer gene topics from the reads. Although several algorithms exist for topic modeling, LDA is preferred in this context as it yields better disambiguation of genes and is less prone to overfitting.^27^^,^^28^^,^^29^

Applying similar techniques to the T cell infiltrate in PDAC represents a logical evolution of these works.^30^ To better understand the dynamics of T cells within PDAC, we used pseudotime analyses and topic modeling of scRNA-seq dataset from 24 PDAC patient surgical specimens,^9^ which has allowed us to identify not just the evolutionary trajectory of various T cells but also the hierarchical importance of which ones to therapeutically target.

## Results

### Single-cell analysis uncovers an immune-suppressive environment during PDAC progression

**Figure 1:**
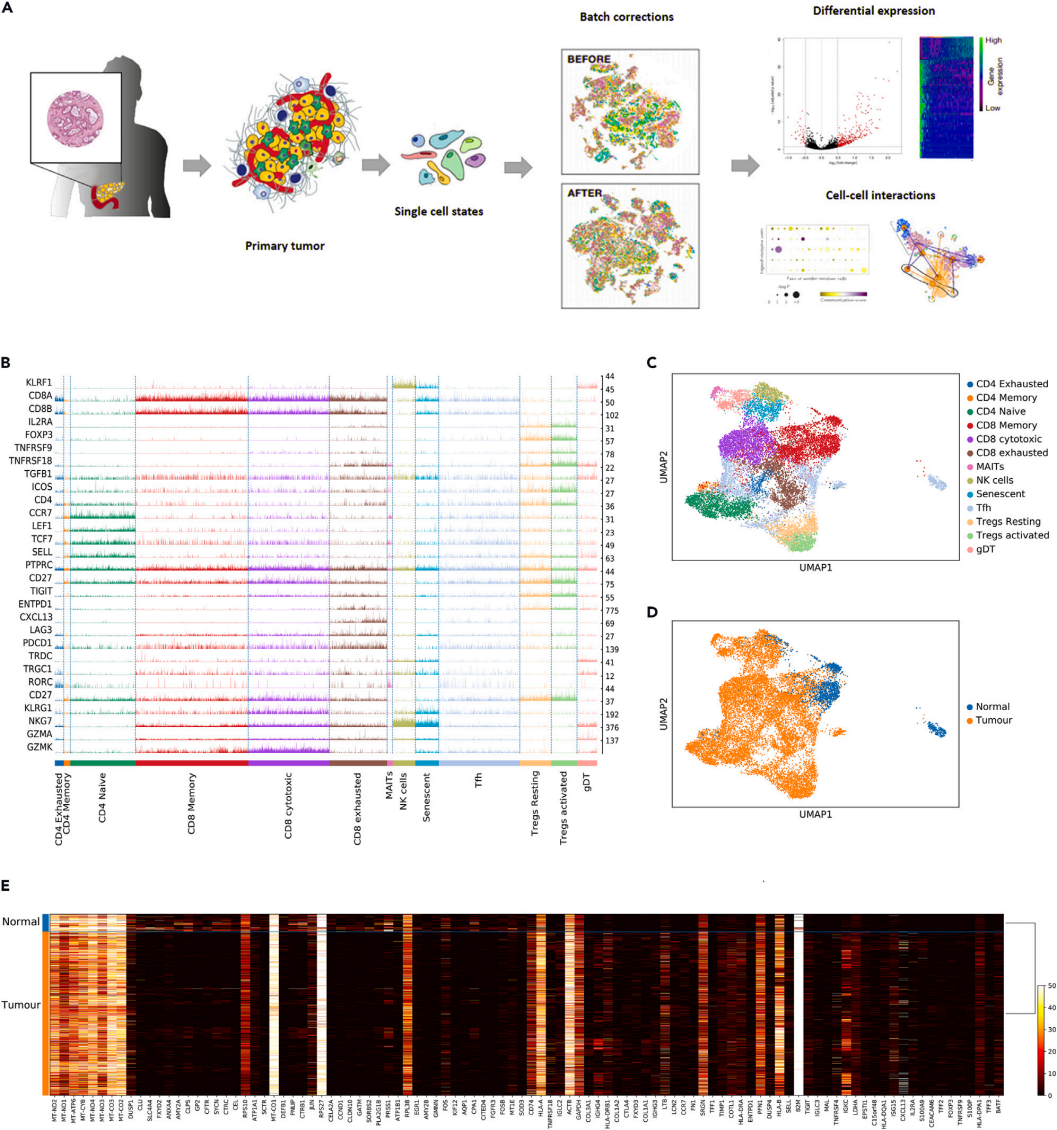
Characterizing the single-cell landscape of T cell biology in PDAC (A) Schematic of the single-cell analysis pipeline: PDAC tumor resection isolated from 24 individuals subjected to cell sorting and single-cell RNA sequencing. The preprocessed gene-cell matrices obtained from the PDAC samples were subjected to PCA, dimensionality reduction, and clustering; Dimension-reduced PDAC dataset is batch corrected and the annotated dataset was prepared for downstream trajectory analysis with scVelo and CellRank. (B) Trackplot illustrating qualitative gene expression profiles of exhausted senescent and cytotoxic T cell subtypes. (C) UMAP dimensionality reduction embedding of the 13k T cells from the combined PDAC transcriptome colored by orthogonally generated clusters labeled by manual cell type annotation. (D) UMAP embedding of the entire dataset colored based on the sample source as normal and tumor. (E) Heatmaps visualization of top 200 differentially expressed genes between different sample sources. It is interesting to see that TNFR, TIGIT, and ENTPD1 are highly expressed in tumor cells, While CLU, FXYD2, CFTR, and CEL are highly expressed in healthy normal cells.

To comprehensively analyze the changes in the landscape of tumor-infiltrating T cells in PDAC, we used a published single-cell RNA-seq dataset.^7^ T cells were subjected to filtering, QC normalization, principal component analysis, batch correction, and downstream trajectory analysis with scVelo and CellRank.^20^ Figure 1A describes this overall workflow as a flow diagram. Signature genes for clustering and annotation of each cluster were in accordance with well-known cell markers recorded in the literature.^7^^,^^9^^,^^31^^,^^32^^,^^33^^,^^34^^,^^35^ Figure 1B illustrates trackplot of qualitative gene expression profiles of different T cell phenotypes including the exhausted, senescent, and cytotoxic T cell subtypes. Figure 1C shows the UMAP dimensionality reduction embedding of the ∼13,500 T cells from the combined PDAC transcriptome colored by orthogonally generated clusters labeled by manual cell type annotation. Subsequently, the PDAC T cell dataset was annotated to identify 13 distinct T cell clusters, including Tregs, CD4^+^, CD8^+^, MAIT, gamma delta T cells, and NK cells. The projection of 24 human PDAC samples based on their source as normal and tumor on UMAP embedding is shown in Figure 1D. It was evident from the differentially expressed genes between different sample sources that TNFR, TIGIT, and ENTPD1 were highly expressed in tumor T cells, while CLU, FXYD2, CFTR, and CEL are highly expressed in healthy normal T cells. Heatmap visualizations (Figure 1E) of the top 200 differentially expressed genes reveal that suppressive and non-tumor-responsive T cells dominate the PDAC T cell microenvironment. This fits with what we and other have previously demonstrated and we set out to investigate the transcriptional programs that drive these T cell phenotypes.^7^^,^^14^^,^^15^^,^^16^^,^^35^

### RNA velocity reveals a conserved hierarchical dynamics in pseudotime lineages of the T cell PDAC transcriptome

**Figure 2:**
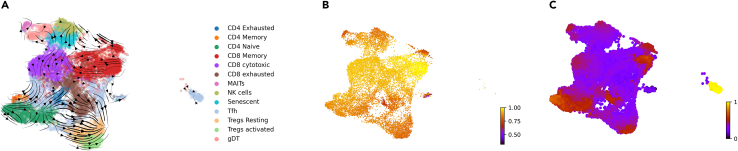
RNA velocity reveals the pseudotime lineages of the T cell PDAC transcriptome (A) RNA velocity vectors projected onto a UMAP embedding using the scVelo package. The velocity vectors indicate a dynamics trajectory profile. The directions indicate likelihood-based evolution characteristics of the expression profile of cells relative to their immediate neighborhood. (B) Random-walk-based implicit velocity pseudotime based on the directed velocity graph. [dark color: start of velocity, yellow: endpoint velocity pseudotime]. (C) Gene-shared latent time based on transcriptional dynamics and representing the cell’s internal clock. The endpoints of the color scale correspond to the origin and end states of velocity the vectors. [ dark color: start of velocity, yellow: endpoint of velocity latentime].

In order to investigate further and look into the PDAC T cell biology, we use RNA velocity, which is a time derivative of the gene expression state based on the abundance of unspliced and spliced mRNAs. RNA velocity provides valuable insight into the cell state trajectories of the T cell subtypes in the PDAC microenvironment. To study the temporal dynamics of T cells in PDAC, RNA velocity vectors were projected onto a UMAP embedding using scVelo (Figure 2A). The directions indicate likelihood-based evolution characteristics of the expression profile of cells relative to their immediate neighborhood. In general, the vector field points from cells with high naive progenitor scores to cells classified to a specific lineage. It was seen that the CD4^+^ and CD8^+^ trajectories initiated from naive populations had diverse end states. The overall velocity projections illustrated multiple incoming and outgoing edges from each cell type clusters and show a complex pseudotime lineage. Figure 2B shows a pseudotime plot which measures the cells' progress through the transition based on the directed velocity graph. It was clear that the CD8^+^ T cells were at the endpoints of most of their transitions (yellow). The gene-shared latent time based on transcriptional dynamics shows the cell’s internal clock (Figure 2C). The cell’s internal clock is the latent time internally accounted for the speed and direction of cell fate and can reconstruct the temporal sequence of transcriptomic events and cellular fates. Therefore, it is an accurate description of the cell’s internal clock, i.e., its position in the underlying biological process. In terms of the cell’s internal clock, most of the PDAC T cells were chronologically in an early state of velocity vector except for the memory and T follicular helper (Tfh) cells. We also probed into the velocity profiles of male and female patients and witnessed a shared trajectory pattern; however, there were differences in the pseudotime dynamics with male samples having significantly higher pseudotime profiles compared to the female ones (Figure S7).

### Partition-based graph abstractions (PAGA) abstraction of pseudotime dynamics indicates a bifurcation of regulatory and cytotoxic T cells

**Figure 3:**
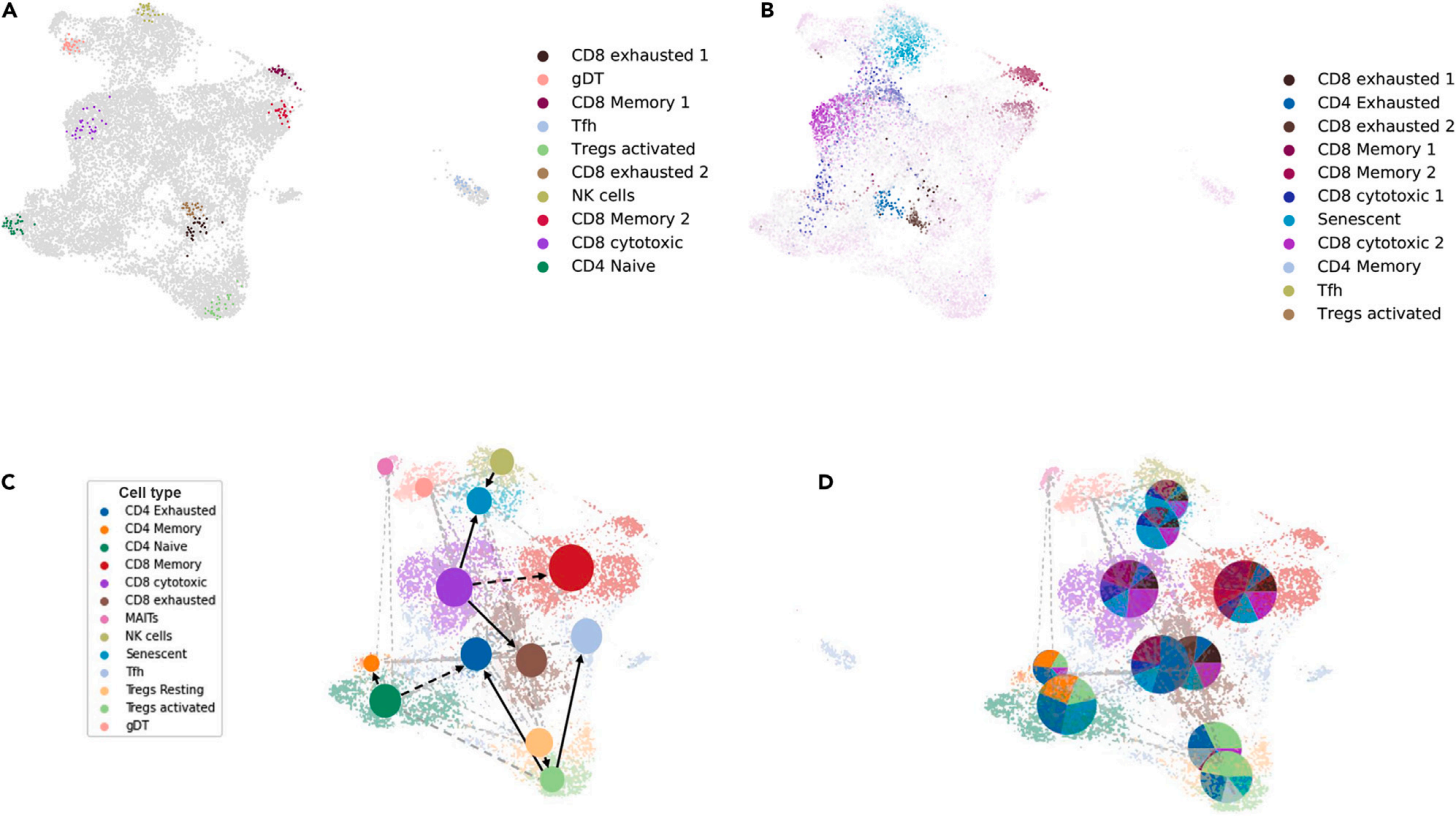
PAGA abstraction of pseudotime dynamics indicate a bifurcation of regulatory and cytotoxic T cells (A) Initial and (B) Terminal macrostates as computed using the Generalized Perron Cluster Cluster Analysis. These computed states are identified with predefined macrostates by coarse-graining the Markov chain into a set of macrostates representing the slow timescale dynamics. (C) Topology preserving PAGA graph superimposed on the UMAP embedding to visualize potential cell type transitions. Nodes represent cell groups and edge weights quantify the connectivity between groups. We see a strong bifurcation of CD8 cytotoxic cells and Tregs into dysfunctional and conventional regulated cell states [See also Figures S1 and S2]. (D) PAGA abstraction with pie charts that show average CellRank fate probabilities corresponding to each of the nodes. A large chunk of cells from all the nodes is seen to evolve toward the senescent and exhausted phase.

To understand the directionality of the biological process involved, CellRank was used to infer initial and terminal populations of a scRNA-seq dataset and compute fate probabilities. Typically, initial macrostates as illustrated in Figure 3A will have low incoming transition probability indicating that it is at the beginning of a cellular lineage, and terminal macrostates (Figure 3B) will have high self-transition probability, indicating a more mature state in the lineage. Cell populations from CD8 cytotoxic, CD4 naive, and Tregs were identified as the initial states for many transitions. Two different subpopulations from CD8-exhausted and CD8 memory cell types were also found to have a low incoming transition probability. Similarly, subpopulations from senescent, Tfh, CD4 memory, and Tregs activated were found to have a high self-transition probability, indicating that they are terminal states corresponding to lineages. Multiple subpopulations from CD8 exhausted and CD8 memory were also found to be terminal states of cell lineages. The fate probabilities computed were then used to infer the coarse-state cellular dynamics using the PAGA (Figure 3B).

The fate probabilities summarized by the PAGA plot were further condensed into pie charts (Figure 3C) revealing exciting insights into the temporal dynamics of the scRNA-seq dataset. We see that cytotoxic CD8 cells have a high transition probability toward the senescent cluster. A major subset of cytotoxic CD8 cells also is closely connected to the exhausted CD8 population. These cluster level fate biases can also be seen in the PAGA pie chart plots. While the CD8 cells transition to exhausted, memory, and senescent, the CD4 naive cells primarily transition toward the memory cell state. A minor portion of the CD4 cells also are transitioning toward the exhausted state. A significant portion of the activated Tregs expression profiles are similar to the expression of CD4-exhausted and Tfh-like functional states. The PAGA graph reinforces these transitions with a pie chart. The pie charts indicate that a significant portion of CD4 naive and CD4 memory is connected to CD4 exhausted and a significant part of CD8 cytotoxic transitioning to the exhausted and senescent states. To further probe into the dynamics of three selected T cell clusters (CD4 exhausted, Tfh, and T_regs_ activated), PAGA abstraction with pie charts was plotted for a subset of the scRNA-seq with these cell types. These results (Figure S1) illustrate a clear functional bifurcation of the activated Tregs trajectories into Tfh and CD4-exhausted T cell macrostates. Furthermore, a recent study by Jacobsen et al.^36^ has also established Tfh cells as a critical player in the germinal center reaction. The study also states that the size of the Tfh cell population closely correlates with that of Foxp3-expressing Treg population. These results are in line with the trajectory observed in Figure S1.

### Topic modeling using LDA reveals different mechanisms of hindrance in PDAC immunity

**Figure 4:**
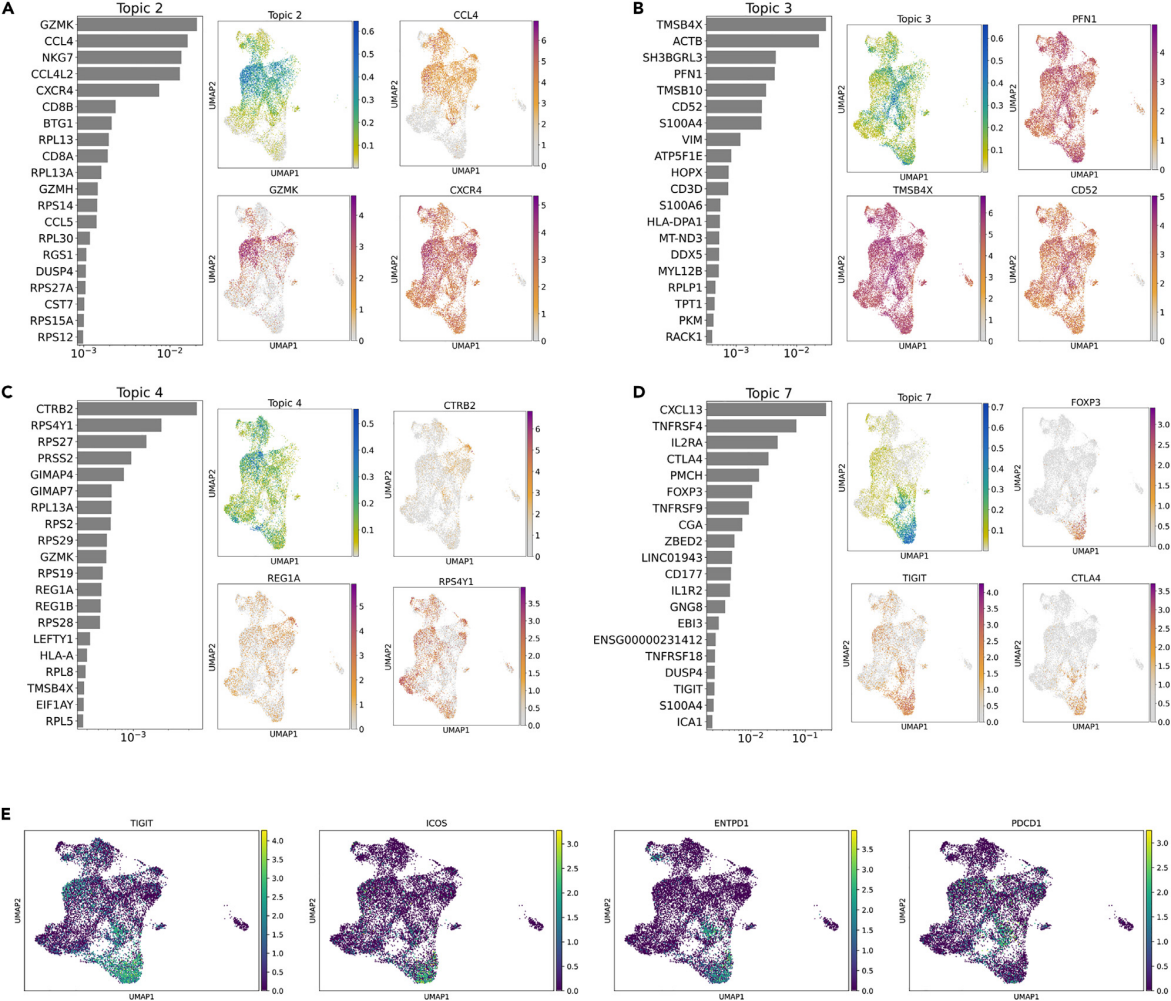
Topic modeling using LDA reveals different mechanisms of hindrance in PDAC immunity Results from topic modeling the T cell transcriptome. Illustrated in figures (A–D) are the top 20 differentially expressed genes for the inferred topics along with an UMAP embedding of the cells colored by topic weight and log-transformed gene expression for the top 3 topic-associated genes. Figure (E) Illustrates the UMAP embedding of scRNA-seq dataset colored by expression profiles of major immune-suppressive and anti-inflammatory genes (TIGIT, ICOS, ENTPD1, and PDCD1).

To investigate transcriptional dynamics in PDAC, we used LDA, a probabilistic grade-of-membership model, to capture continuous and composite states. LDA represents each cell as a mixture of *de novo* inferred “topics” or programs, modeled as distributions over genes. Our 10-topic model coherently captured distinct functional programs. In contrast to focusing on individual cell identities, analyzing the T cell landscape from the perspective of topics gives an oversight of immune mechanisms in action in the tumor microenvironment and allows for a more informed targeting of specific pathways to improve antitumor responses. Topic 2, “Cytotoxic”, highlights genes characteristic of cytotoxic and memory CD8 T cells and those associated with chemokine signaling like CXCR4 and CCL4 (Figure 4A). Topic 3, “Structure and mobility”, features genes associated with exhausted and Tfh cells and actin cytoskeleton remodulating genes ACTB, PFN1, and CD52 (Figure 4B). Topic 4, “Proliferative” enriched in CD8-associated genes (CTRB2, RPS4Y1, REG1A, PRS27, and GZMK) (Figure 4C); cells scoring high in topic 4 also preferentially expressed PRSS2, which encodes serine protease 2.^37^ Topic 7, “Inhibitory”, includes Tregs-associated genes (IL2RA, CLTA4, FOXP3, and TIGIT) (Figures 4D and 4E). Topics 5 and 6 feature other immune functions (for example, stress responses and mitochondrial translation).^38^ Topics 3 and 8 were associated with functional classes of T cells while Topic 9 resembles those of natural killer (NK) cells or terminally differentiated/NK-like T cell states. Some topics (1, 7, 9, and 10) are dominated by essential cellular functions and biological processes. Even though, the velocity analysis pointed to transience of the functional states, and the topic modeling picked out functional gene expression patterns (Figure S5). These functional states are essentially transient and therefore are not effective.

## Discussion

In this study, the trajectory profiles obtained from the scVelo generalized dynamic model of RNA velocity indicate that there are distinct velocity patterns even among cells belonging to a specific T cell subtype. T cell subclusters had a non-coherent trajectory profile and did not have a unified trajectory. To have a coarse-grained look into these multifaceted trajectories, we resorted to partition-based graph abstraction (PAGA) to obtain an interpretable graph-like map of the arising data manifold. The PAGA graph represents the T cell subtypes as nodes and assigns widths for incoming and outgoing edges based on transition probabilities.

The PAGA graph revealed interesting insights into the behavior of cytotoxic CD8 and Tregs in the PDAC microenvironment. A distinct bifurcation profile was observed in the cytotoxic CD8 T cell and Treg trajectories. In the case of Tregs, the dominant destination states were exhausted and functional Tfh-like states (Figure 4E). However, in the case of cytotoxic CD8 cells, memory-like or senescent-like expression profiles were the dominant bifurcation directions. This bifurcation in trajectory indicates that cells of a given cell type were able to behave distinctly. In an attempt to further understand this bifurcation and to explore avenues to control the trajectory, we investigate the genes which are driving this trajectory (Figures S2 and S3).

In order to capture continuous and composite states across cell states, we used a LDA. This probabilistic grade-of-membership model represents each cell as a mixture of *de novo* inferred “topics” or programs, modeled as distributions over genes. The versatility of this model allows it to understand how a cell state might vary within a given cell type. The LDA assigns a representative set of gene topics to each cell and these topics defined by the expression profiles of the genes might not be an exhaustive profile. This helped us understand why we observed a multitude of cell states within each cell type. Topic modeling helped us identify 10 different topics throughout the T cell microenvironment in PDAC. Topic 7 resembled inhibitory and regulatory while Topic 8 represented a functional-like/activated T cell profile. CXCL13 which dominated topic 7 has been shown to indicate neo-antigen reactive T cells.^39^ This showed the overlap between regulatory cells and tumor-reactive cells, as most of the reactive cells were Tregs. CXCL13 has also been seen in TLS formation thereby validating the Tregs transitions to Tfh-like functional state. Topic 3 seemed to be more innate like with S100 proteins and actin can be an innate activator through DNGR-1. Even though both topics 2 and 3 represented cytotoxic and activated-like T cell states, topic 2 seems likely to be more senescent and terminal due to the dominance of GZMK in comparison to topic 3. Interestingly, genes associated with pancreatic cancer such as MALAT1 (noncoding RNA), CTRB2 (serine protease), CST7 (cysteine peptidase inhibitor), and BTG1 (anti-proliferation factor) were picked in multiple topics. These topics shed light on the various mechanisms which cause hindered immunity in the PDAC microenvironment. The topic profile of cells within the Tregs and CD8 clusters was found to be composed of two major topics suggesting that these topics might be responsible for the bifurcation in the trajectory analysis. Upon looking into the genes described by these topics, it was seen that the Tregs were dominated by immune-suppressive genes and functional topics while the CD8 clusters have topics with senescence and exhaustion-related genes dominating them. It has to be noted that genes like MALAT1 are known to be indicative of cell health. Therefore, it should be acknowledged that a few topics may reflect technical factors.

In this work, we describe a novel model of cell states as a behavioral phenotype within a cell type, even though the cell type dictates the overall gene expression of the cells. The splicing dynamics from RNA velocity and topic modeling help us understand the fine-grain differences in gene expression which make every cell unique. These unique characteristics modeled by the LDA may arise from the distinct spatiotemporal distribution of the cells in the PDAC microenvironment. These spatiotemporal factors shape their cell state and define the topic which the cell expresses. For instance, a T cell that is localized in an environment with a high concentration of stromal or epithelial cells will be influenced by the former’s secretions. We propose that these interactions highly influence the cell state of the given T cells.

In conclusion, topic modeling is an unbiased way of looking into the complex true fate of the cell. The results from topic modeling in our analyses are in line with the cellular annotation-based scVelo and PAGA trajectories. This validates the potential of the topic modeling to investigate cell fates. We truly believe that the work is novel and this platform will be used to develop new drugs which can help manipulate the different trajectories of the cell types and thereby govern their functional phenotypic states.

### Limitations of the study

This study is an in-depth analysis of the pseudotime characteristics of a comprehensive T cell scRNA-seq data from PDAC samples. However, it still is limited by the patient demographic of the dataset. To address this, we incorporated data from the reference scRNA-seq dataset published by Chijimatsu et al. The slingshot analyses from the combined dataset supported the bifurcation of immune trajectories into suppressive and non-tumor-responsive T cells. Although trajectories from the combined dataset reinforce our results, future work involving velocyto analysis of larger dataset will be needed to understand the intricacies of the trajectories involved.

## STAR★Methods

### Key resources table

**Table:** 

REAGENT or RESOURCE	SOURCE	IDENTIFIER
**Deposited data**		

Raw PDAC scRNAseq	Peng et al. 2019^9^	CRA001160
PDAC reference dataset	Chijimatsu et al., 2022^46^	zenodo [https://doi.org/10.5281/zenodo.6024273]

**Software and algorithms**		

iPython notebooks and R code used for analysis	This paper	https://doi.org/10.17605/OSF.IO/8TF4W
Seurat v4.2.0	Hao et al. 2021	https://satijalab.org/seurat/
scvelo v0.2.3	Bergen et al. 2020^21^	https://github.com/theislab/scvelo
CellRanger (v3.1.0)	10x Genomics	Zheng et al. 2017^25^
CellRank v1.2.0	Theis et al. 2020	https://github.com/theislab/cellrank
LDA topic modeling	Bielecki et al. 2021^29^	https://github.com/klarman-cell-observatory/skin-ILCs
Slingshot v2.0.0	Street et al. 2018^45^	https://bioconductor.org/packages/release/bioc/html/slingshot.html
R v4.1.0	R Core	https://www.r-project.org/
Python v3.7.0	Python Software Foundation	https://www.python.org/

### Resource availability

#### Lead contact

Further information and requests for resources should be directed to and will be fulfilled by the lead contact, Shivan Sivakumar (shivan.sivakumar@oncology.ox.ac.uk).

#### Materials availability

This study did not generate any new materials.

### Method details

#### Data characteristics

The fastq reads for the single-cell transcriptome analysis were obtained from the NGDC GSA (Genome Sequence Archive) database (GSA: https://bigd.big.ac.cn/gsa), under accession number CRA001160. The article corresponding to the repository illustrates that all the PDAC patient samples were obtained from the Department of General Surgery of Peking Union Medical College Hospital (PUMCH). In total, 24 samples of PDAC tissue were obtained from the database, along with 11 samples of normal pancreatic tissue. This study conforms with the ethical guidelines (Declaration of Helsinki) and has secured approval from the Ethics Committee of Peking University First Hospital (Approval No. 2019-147). Further, the article states that written informed consent was obtained from all participants.

#### Pre-processing and annotating T-cell subtypes

The fastq reads obtained from GSA were processed using the CellRanger (v3.1.0) counts pipeline using the recommended parameters.^40^^,^^41^^,^^42^ The cell count matrices were obtained by aligning the sequencing reads with the Human GRCh38-3.0.0 transcriptome as the reference. These filtered gene–cell matrices were then read into the Seurat v4.0 pipeline implemented in R for downstream analysis.^43^ The standard Seurat preprocessing workflow was followed with QC based cell filtration, data normalization and scaling. Low-quality cells with less than 200 unique feature counts and cells with a mitochondrial count greater than 5% were excluded.

Further, Seurat was used to identify major cell types with highly variable genes. Following this, Significant principle components were determined using JackStraw analysis and visualization of heatmaps focusing on PCs 1 to 20. PCs 1 to 15 were used for graph-based clustering to identify distinct groups of cells. These groups were then projected onto the UMAP embedding computed using the previously calculated principle components. Known marker genes were used to characterize the identities of the cell types.

#### Velocity and pseudotime analysis

Velocyto (0.6) was used to estimate the spliced and unspliced counts from the pre-aligned bam files42. RNA velocity, latent time, root, and terminal states were calculated using the dynamical velocity model from scVelo (0.2.2)43. Kendall’s rank correlation coefficient (τ) was used to correlate the expression patterns of biologically significant genes with latent time. The counts matrices from CellRanger was input into the velocyto pipeline to predict cell fates. Velocyto CLI was used to estimate the spliced-unspliced count from the 10x runs. The loom files generated were then merged into the Seurat object for pseudotime analysis in python. RNA velocity latent time, root, and terminal states were calculated using the scVelo package implementation in Scanpy 1.9.1.^44^

The scv.pp.filter_and_normalize function was used to filter, normalize and preprocess the annotated data. The gene-specific velocities were computed from the spliced and unspliced abundances using scv.tl.velocity(mode = ‘dynamic’) and scv.tl.velocity_graph() functions. These velocities were visualized over the precomputed UMAP embedding using scv.pl.velocity_graph() or scv.pl.velocity_embedding_grid() functions. To better characterize the topology of the dataset and inter-cluster relationships between cells, partition-based graph abstraction (PAGA) was used. PAGA parameters were calculated and visualized using sc.tl.paga(), sc.pl.paga(), sc.tl.draw_graph(init_pos = ‘paga’), sc.tl.dpt(). The final Scanpy object was subject to Cellrank analysis for the reconstruction of cell state dynamics using directional information from RNA velocity. The pl.cluster_fates within CellRank was used in the ‘paga-pie’ setting to visualize the fate probability of each cell population. Further, the initial and terminal states were visualized using the pl.initial_states and pl.terminal_states functions from the cell rank module. Further, we observe that there are different subsets of CD8 Cytotoxic and Memory cells which are classified as terminal/initial states. To probe into the difference between these subsets, a list of driver genes corresponding to each of these subsets were generated and are provided as Table S1. These tables also contain their p value, corrected p value and the 95% confidence interval for the correlation statistic.

In addition to these tables, a table of top 300 driver genes driving the bifurcation into functional and dysfunctional states were obtained from their Sorted velocity fit likelihood and is provided as Table S2. Further, the Slingshot module implemented in R was used to validate the pseudotime–based cell trajectory analysis.^45^ The slingshot wrapper function was used with the UMAP dimensionality reduction and cluster labels as in Seurat objects to identify the trajectory. The slingshot trajectories were calculated such that each trajectory has a single start and end point. For clarity and brevity, multiple trajectories were plotted on the same graph (Figure S4).

In an attempt to further validate the pseudotime trajectory results from Scvelo, We have expanded our dataset with the PDAC scRNA-seq atlas from Chijimatsu et al.^46^ which contains 130,000 cells from six datasets and 70 samples. The cells in the combined dataset were annotated and slingshot was performed to validate the results. The plots from the analyses of the combined dataset are provided as Figure S6. The difference in velocity profiles between male and female patients were probed by plotting velocyto, latent-time, pseudotime (Figure S7), and PAGA (Figure S8) graphs. In an effort to compare the velocity trajectory of the T - cells from PDAC with the healthy T – cells, we calculated the velocity embeddings, latent time and pseudotime (Figure S9) for the healthy cells from Peng et al. However, it must be noted that we were not able to plot the PAGA due to the lack of a satisfactory cell count. A lower cell count per cluster translates into a weak velocity signals. This in turn makes unreliable predictions about the underlying topological information from RNA velocity.

#### Topic modelling

Topic modelling results were obtained by fitting a ‘grade-of-membership’ LDA model to the raw counts matrix. The matrix was subject to filtration of low-quality cells using the parameters mentioned in the above paragraph. The CountClust Bioconductor package (v1.12.0) was used to fit the LDA Model to the scRNAseq data. The models were fitted with a range of different arguments to the maximum number of topics parameter, ranging from K = 5 to K = 20. The optimal number of topics was chosen to be K = 10 using the minimum for the Bayesian information criterion (BIC). A minimum for the BIC indicates that a balance is struck between capturing systematic signals and preventing overfitting. The top genes corresponding to the topics were extracted via CountClust’s ExtractTopFeatures function. Gene sharing was allowed between clusters, and top 200 genes were selected for each of the clusters. ExtractTopeatures was implemented using the Poisson model for the Kullback-Leibler (KL) divergence by maximizing the minimum KL divergence of a topic. Selected genes were then ranked by sorting them in descending order of their minimum for KL Divergence. The top 200 genes were then selected from the ranked gene list for each of the topics.

### Quantification and statistical analyses

All analyses were performed using the dynamical velocity model from scVelo (0.2.2)^43^ package and hence the statistical tests incorporated by scvelo were used. Scvelo uses a likelihood-based dynamical model to model transcriptional dynamics. For each gene in the transcriptome, scVelo computes a likelihood to model-optimal latent time and transcriptional state. This likelihood factor estimates how well a cell is described by the learned spliced/unspliced phase trajectory. The genes with a very high likelihood are designated to be driver genes – genes that are responsible for the velocity transition. scVelo further incorporates the likelihood in its dynamic model by minimizing negative log-likelihood through the modelling process. For further information, please refer to the scvelo article.^43^ The PAGA algorithm on the other hand uses a statistical model for the connectivity among a groups of cells. This connectivity is computed through graph-partitioning or alternatively through clustering or experimental annotation. The statistical tests used in PAGA is described in detail in the article introducing graph abstraction as a topology preserving map of cells.^47^

## Data Availability

No original data was generated in this study. The code used to analyse all the data have been deposited with The Open Science Framework (https://doi.org/10.17605/OSF.IO/8TF4W) and are publicly available as of the date of publication. DOIs are listed in the key resources table. Any additional information required to reanalyse the data reported in this paper is available from the lead contact upon request.

## Supplemental information

Document S1. Figures S1–S9

Table S1. Table of genes driving the velocity profiles of cytotoxic, memory and exhausted T cells, related to Figure 3; see also Figure S1 Since the velocity subpopulations are based on the velocity profile and not gene expression, we cannot provide differentially expressed genes. We however can provide the driver genes for each of these subpopulations. These driver genes have an upregulated splicing ratio and are responsible for the velocity subpopulations. They are provided as a supplementary table 1 with their associated correlation, p value, corrected p value and the 95% confidence interval for the correlation statistic.

Table S2. A table of the top 300 genes driving velocity profiles, related to Figure 2 A table of top 300 driver genes driving the bifurcation of velocity profiles into functional and dysfunctional states as obtained from the Sorted velocity fit likelihood.
