# Supplementary material for: Pseudotime dynamics of T cells in pancreatic ductal adenocarcinoma inform distinct functional states within the regulatory and cytotoxic T cells
Source: iScience. 2023 Mar 7;26(4):106324. doi: 10.1016/j.isci.2023.106324 (PMC10034436; doi:10.1016/j.isci.2023.106324)
Supplement: Document S1. Figures S1–S9 [file mmc1.pdf]

iScience, Volume *26*

Supplemental information

Pseudotime dynamics of T cells in pancreatic ductal adenocarcinoma inform distinct functional states within the regulatory and cytotoxic T cells

Ashwin Jainarayanan, Nithishwer Mouroug-Anand, Edward H. Arbe-Barnes, Adam J. Bush, Rachael Bashford-Rogers, Adam Frampton, Lara Heij, Mark Middleton, Michael L. Dustin, Enas Abu-Shah, and Shivan Sivakumar

SUPPLEMENTARY FIGURE 1

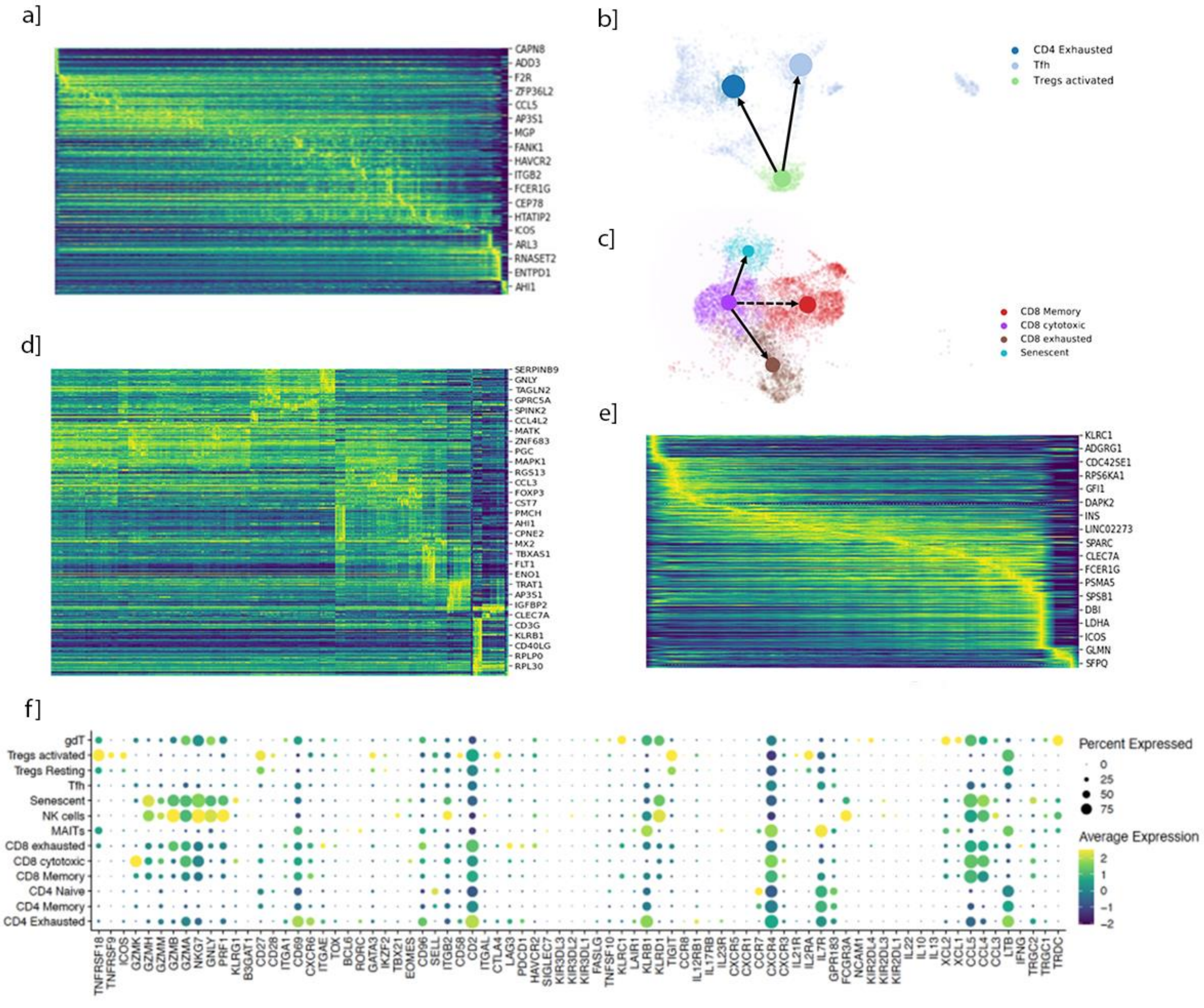

SUPPLEMENTARY FIGURE 2

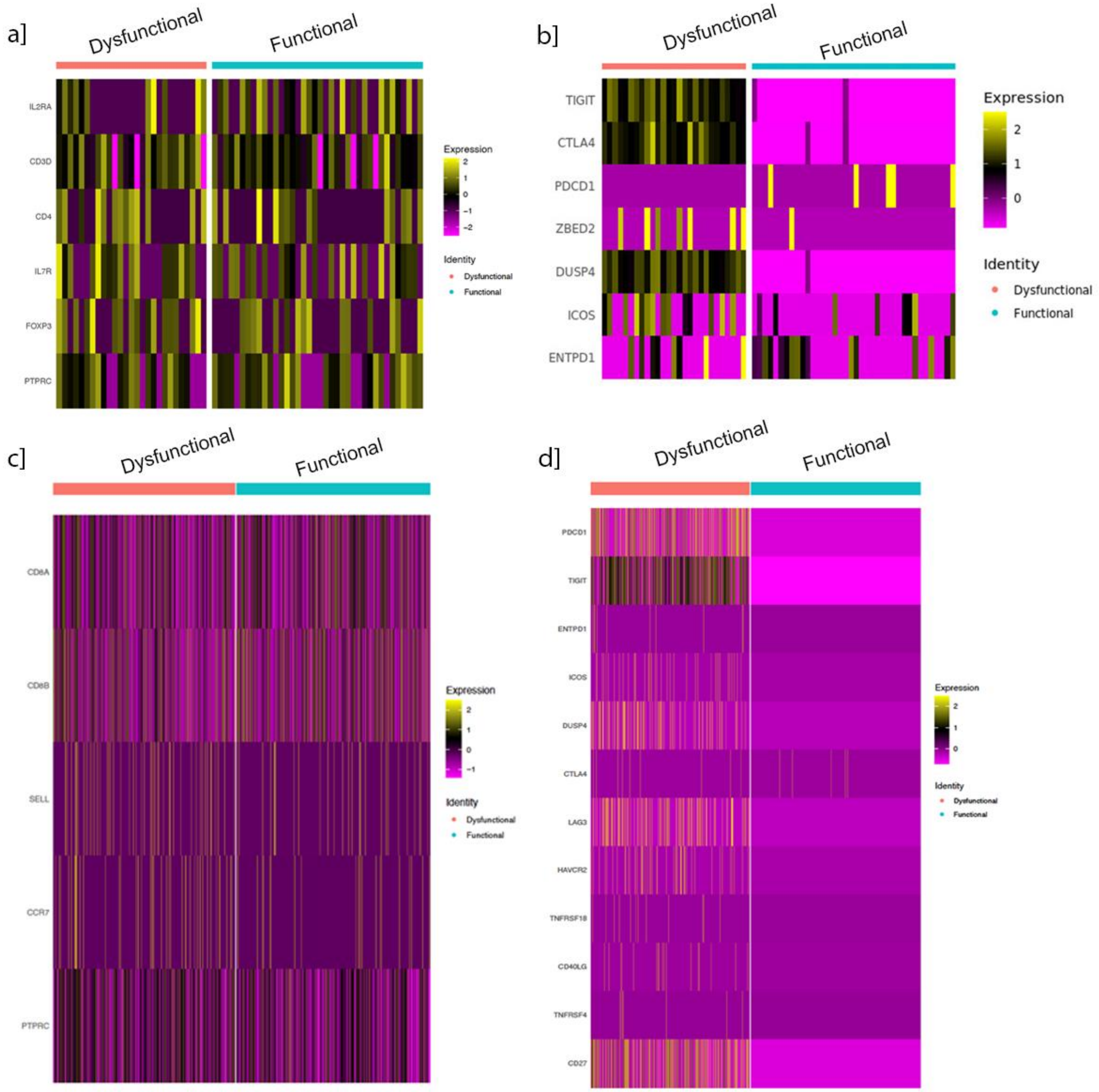

SUPPLEMENTARY FIGURE 3

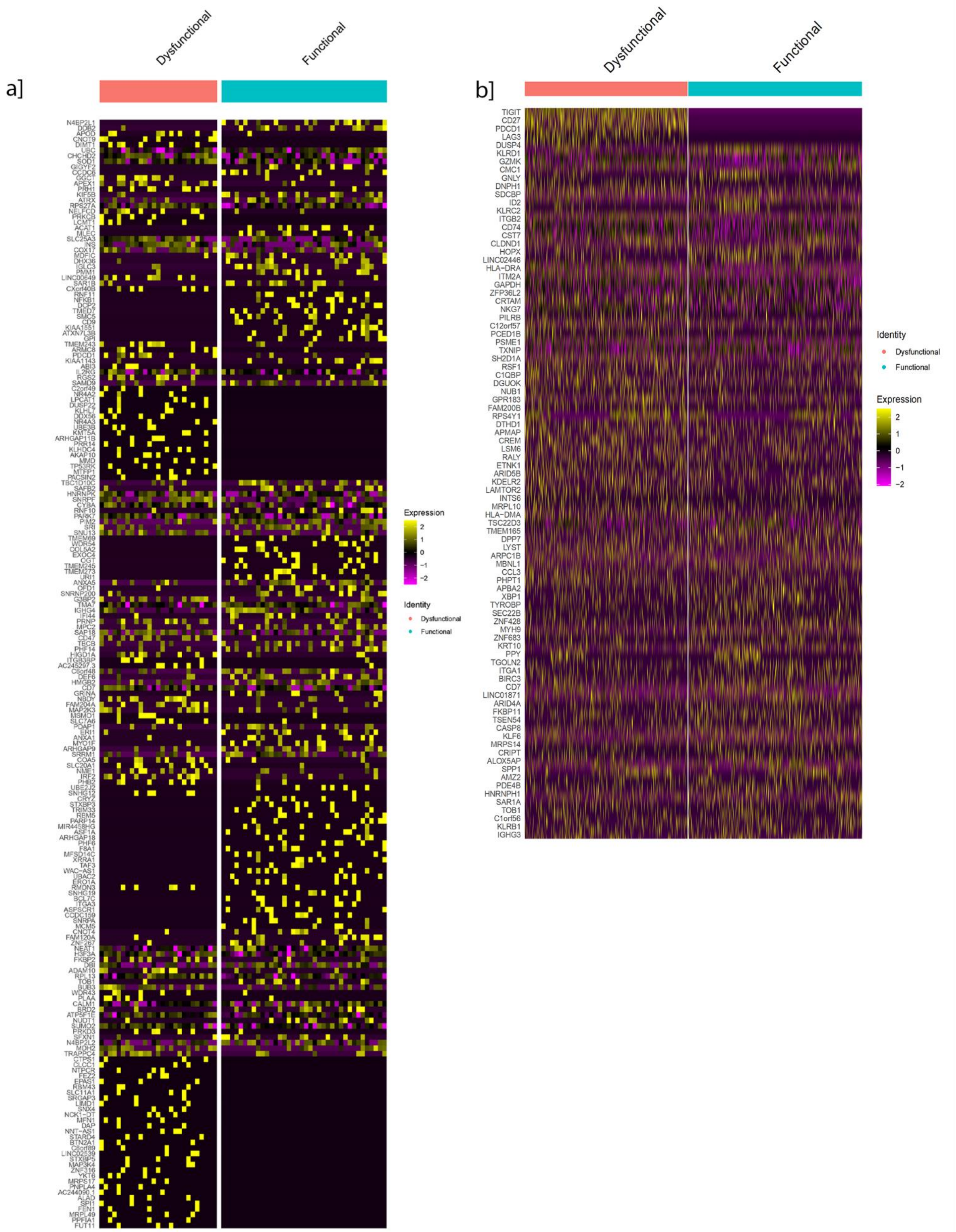

SUPPLEMENTARY FIGURE 4

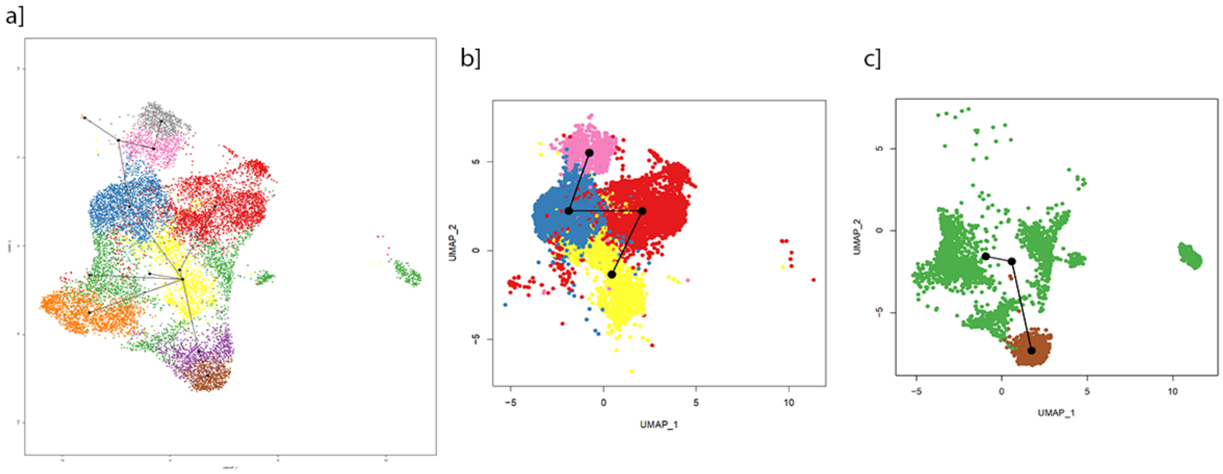

SUPPLEMENTARY FIGURE 5

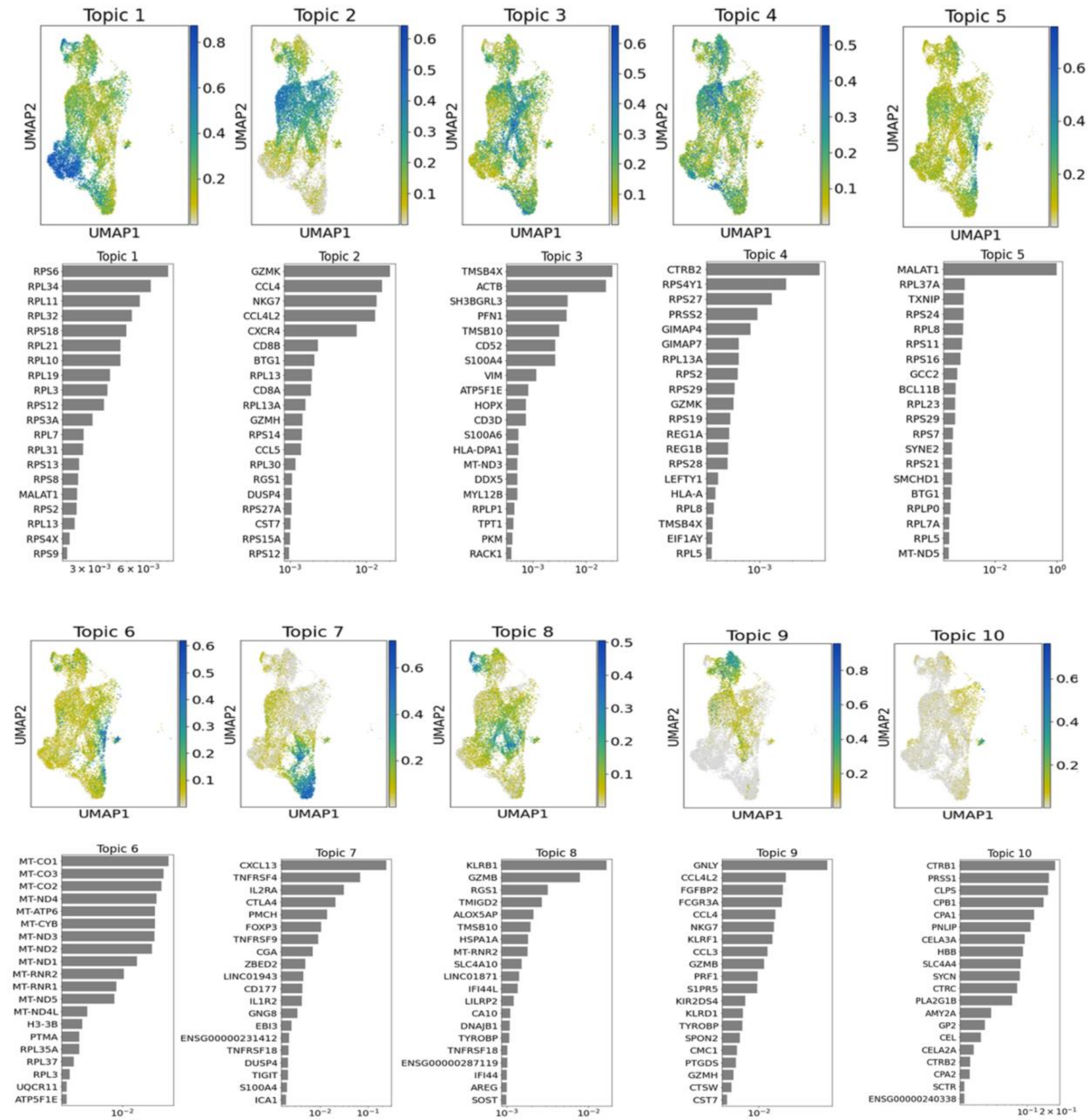

SUPPLEMENTARY FIGURE 6

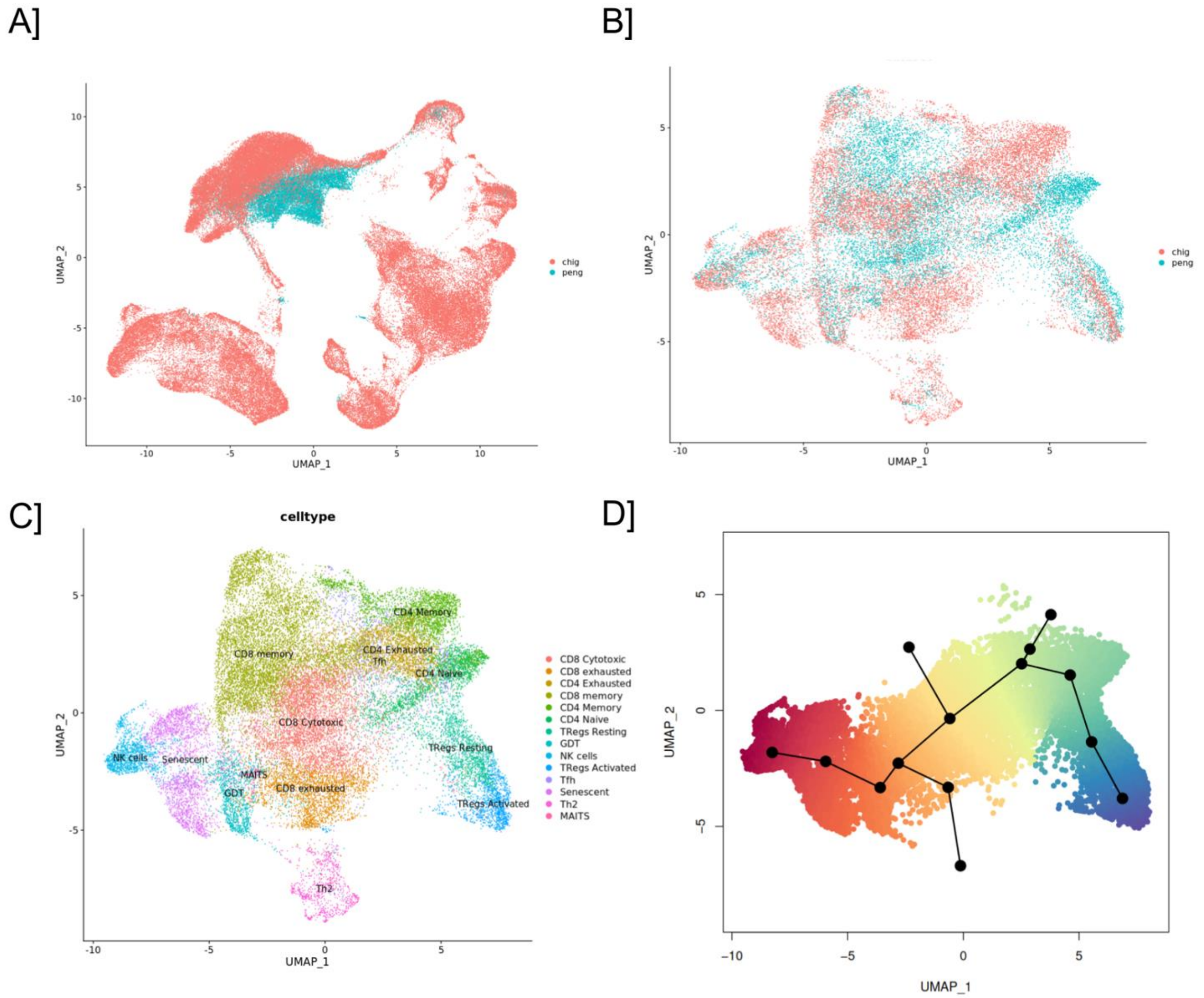

SUPPLEMENTARY FIGURE 7

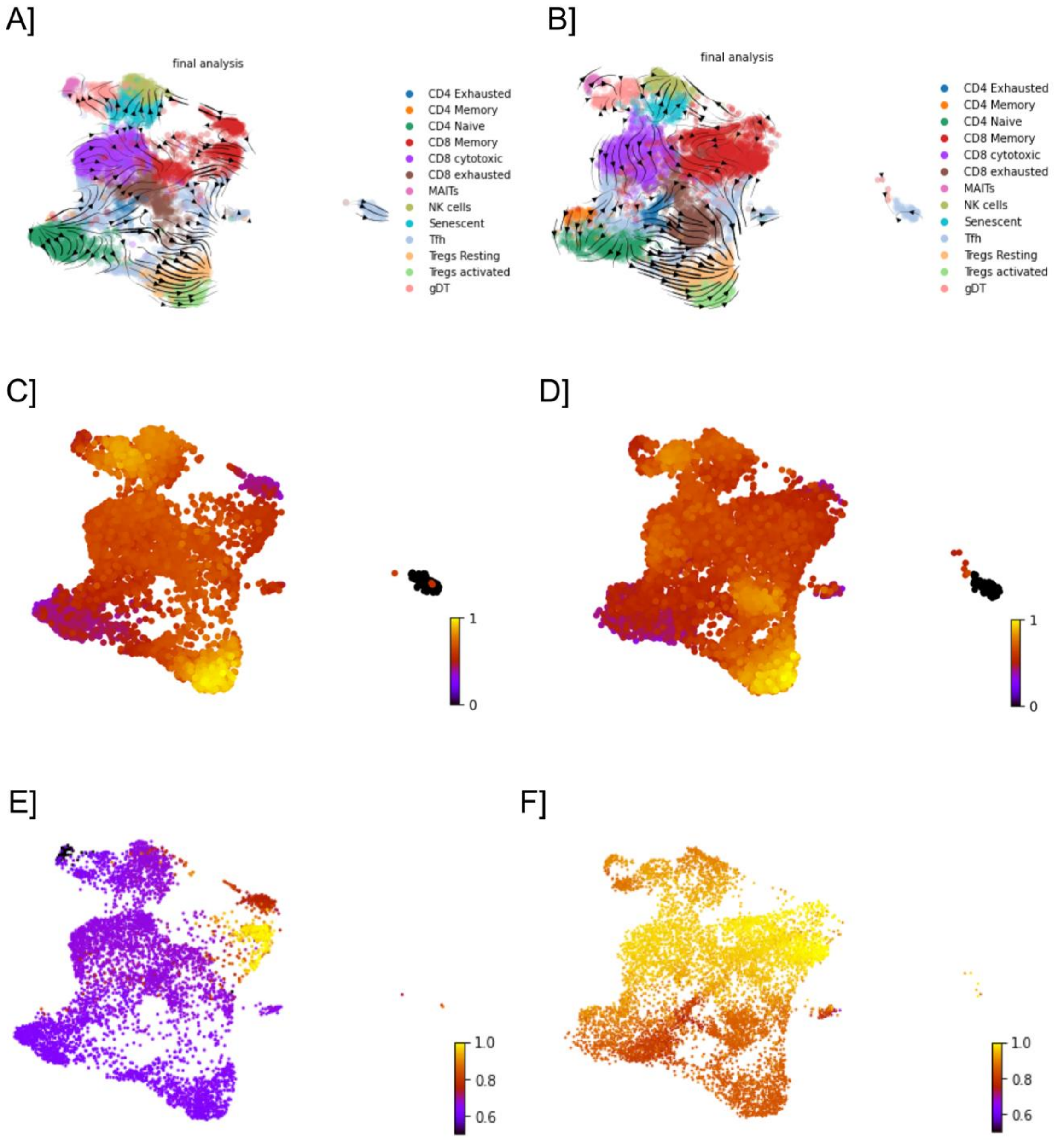

SUPPLEMENTARY FIGURE 8

A]

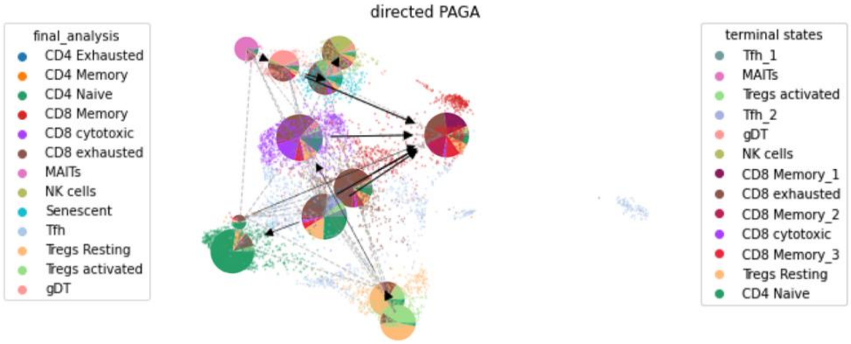

B]

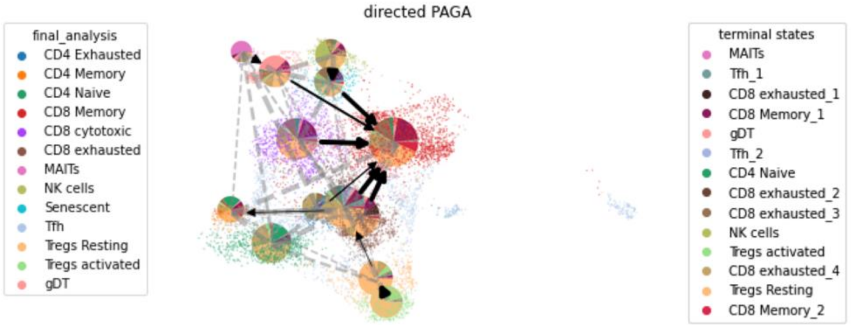

SUPPLEMENTARY FIGURE 9

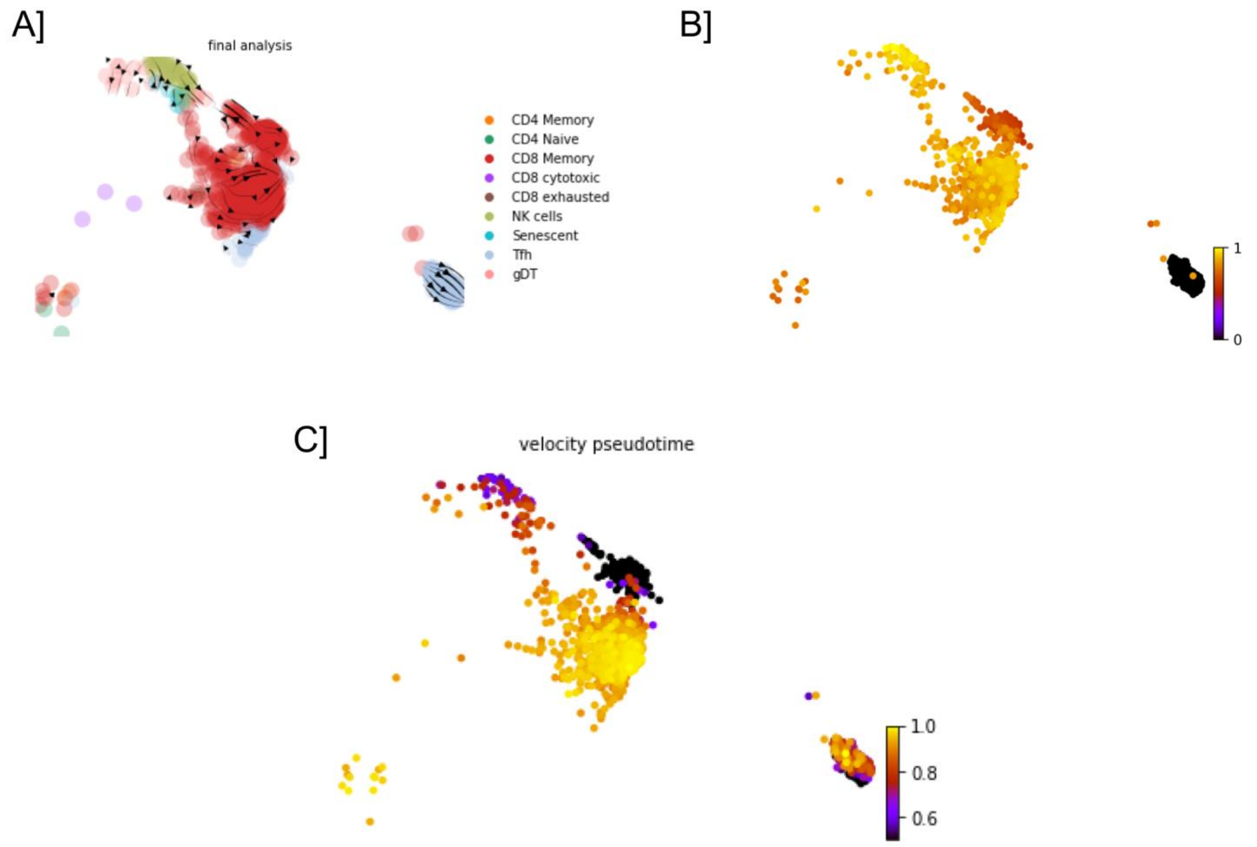

FIGURE LEGENDS

SUPPLEMENTARY FIGURE 1: Velocity Dynamics of selected T-cell clusters (CD4 Exhausted, Tfh and Tregs Activated). Related to figure 3

a] Heatmap depicting the expression profile of the top genes sorted by fit likelihood recovered from the dynamical latent time model [S1]. The X-axis denotes the increasing value of latent time from the left to right. Several genes such as ENTPD1, ICOS that are responsible for the immune-suppressive and anti-inflammatory activity of regulatory T-cells are expressed along the latent time axis. These regulatory pathways prevent the immune system from inhibiting the progression of cancer. (Supplementary Table 1)

b] The PAGA abstraction for the three selected T-cell clusters (CD4 Exhausted, Tfh and Tregs Activated) illustrates a clear bifurcation of the activated Tregs into Tfh and CD4 Exhausted T-cell macrostates [S2].

c] The PAGA abstraction for a subset of the T-cell transcriptome with CD8 Memory, CD8 Cytotoxic and CD8 Exhausted cells illustrates differentiation trajectory of cytotoxic CD8 T-cells into exhausted, senescent and memory phases.

d] Heatmap depicting the expression profile of the top genes for the selected T-cell subset illustrated in figure b] sorted by fit likelihood recovered from the dynamical latent time model. X-axis denotes increasing value of latent time from the left to right.

e] Heatmap depicting the expression profile of the top genes for a subset of the T-cell transcriptome illustrated in figure c] sorted by fit likelihood recovered from the dynamical latent time model. X axis denotes increasing value of latent time from the left to right.

f] Dot plot depicting per cent expression and average expression of key genes involved in identifying the phase profile of the T cells. Suppressive and non-tumour responsive genes predominate T cells PDAC landscape

SUPPLEMENTARY FIGURE 2: Differential gene expression heatmap of different cell clusters. (Related to figure 3)

a] Heatmap depicting the indifference in Treg marker gene expression profiles between the functional and dysfunctional states.

b] Heatmap depicting the key difference in expression profiles between the functional and dysfunctional states of Tregs derived from the pseudotime analyses.

c] Heatmap depicting the indifference in cytotoxic CD8 marker gene expression profiles between the functional and dysfunctional states.

d] Heatmap depicting the key difference in expression profiles between the functional and dysfunctional states of cytotoxic CD8 derived from the pseudotime analyses.

SUPPLEMENTARY FIGURE 3: Differential gene expression heatmap of different velocity profiles. (Related to figure 2)

a] Heatmap depicting the differential gene expression profiles between the functional and dysfunctional states of Tregs.

b] Heatmap depicting the differential gene expression profiles between the functional and dysfunctional states of cytotoxic CD8s.

SUPPLEMENTARY FIGURE 4: Slingshot analyses of the PDAC dataset (Referenced in STAR methods)

a] Slingshot trajectories of the T cell transcriptome is illustrated in fig a].

b] Figure b] illustrates the slingshot trajectories computed from the three selected T-cell clusters (CD4 Exhausted, Tfh and Tregs Activated).

c] Figure c] illustrates the slingshot trajectories computed from a subset of the T-cell transcriptome with CD8 Memory, CD8 Cytotoxic and CD8 Exhausted cells.

SUPPLEMENTARY FIGURE 5: Characteristic genes from the LDA topics indicate suppressive and non-tumour responsive T-cells predominate PDAC. (Related to figure 4)

LDA model results for the rest of the topics. For each topic, the UMAP embedding of scRNA-seq profiles is coloured by topic weight. Below each of the embeddings is a bar plot of top-scoring genes ranked by a logarithmic score of association with the particular topic.

SUPPLEMENTARY FIGURE 6: Annotation and Slingshot analysis of the larger PDAC dataset from Chigimatsu et al.[S3] (Related to STAR methods)

A] is a UMAP of the cells obtained from Peng et al and the cells obtained from the reference dataset: Chigimatsu et at. Figure B] is the Harmonized UMAP of just the T cells from both the

dataset coloured by their source [S3]. Figure C] Illustrates the cell types annotated on the combined dataset UMAP. The Slingshot trajectory of the combined dataset is shown in figure D]. We can see that a trajectory similar to the velocyto profile towards the senescent states is seen.

SUPPLEMENTARY FIGURE 7: Difference in velocity profile between male and female patients (Related to figure 2)

A], C] and E] illustrates the velocity embeddings, the latent time plot and the pseudotime map of the male patients from Peng et al while figures B], D] and F] illustrate the same for the female patients from Peng et al. The difference between males and females is negligible in the velocity and latent time plots. However there is a significant difference in pseudoptime computed between male and female patients. It is difficult to draw a conclusion from these differences first due to the low number of samples and second due to inherent immunological differences between the male and female immune system which could be a confounding factor.

SUPPLEMENTARY FIGURE 8: Difference in PAGA profiles between male and female patients (Related to figure 2)

A] Illustrates the PAGA graph abstraction for male patients while figure B] illustrates the PAGA plot of cells from female patients. The broad PAGA profile remains similar in the male and female subsets. However one could see a stronger convergence towards the CD8 memory phenotype in females compared to males. This could stem from the inherent immunological differences between male and female patients.

SUPPLEMENTARY FIGURE 9: Difference in velocity profile between healthy and PDAC patients (Related to STAR methods)

The velocity, latent time and pseudotime profiles of healthy cells from Peng et al are illustrated in A], B] and C] respectively (Referenced in STAR methods). It is evident from the plots that the healthy cell count is lower compared to tumour cells count. A lower cell count per cluster translates into a weak velocity signals. This in turn makes unreilable predictions about the underlying topological information from RNA velocity.

SUPPLEMENTAL REFERENCES:

1. Bergen, V., Lange, M., Peidli, S., Wolf, F. A., and Theis, F. J. (2020). Generalizing RNA velocity to transient cell states through dynamical modeling. *Nature Biotechnology*, *38*(12), 1408-1414. doi:10.1038/s41587-020-0591-3

2. Wolf, F.A., Hamey, F.K., Plass, M., Solana, J., Dahlin, J.S., Göttgens, B., Rajewsky, N., Simon, L., and Theis, F.J. (2019). PAGA: Graph abstraction reconciles clustering with trajectory inference through a topology preserving map of single cells. Genome Biology*20*.

3. Chijimatsu, R., Kobayashi, S., Takeda, Y., Kitakaze, M., Tatekawa, S., Arao, Y., Nakayama, M., Tachibana, N., Saito, T., Ennishi, D., et al. (2022). Establishment of a reference single-cell RNA sequencing dataset for human pancreatic adenocarcinoma.Iscience *25*, 104659.
